# TOP2A Promotes Tumorigenesis of High-grade Serous Ovarian Cancer by Regulating the TGF-β/Smad Pathway

**DOI:** 10.7150/jca.42736

**Published:** 2020-04-25

**Authors:** Yan Gao, Hongyu Zhao, Meng Ren, Qi Chen, Jie Li, Zhefeng Li, Chenghong Yin, Wentao Yue

**Affiliations:** 1 Central Laboratory, Beijing Obstetrics and Gynecology Hospital, Capital Medical University, Beijing 100026, China; 2 Departments of Internal Medicine, Beijing Obstetrics and Gynecology Hospital, Capital Medical University, Beijing 100026, China

**Keywords:** high-grade serous ovarian cancer, topoisomerase IIα, TGF-β/Smad, DEGs

## Abstract

**Background:** High-grade serous ovarian cancer (HGS) is the most aggressive form of ovarian cancer due to its rapid spread, insidious onset, and early dissemination throughout the abdominal cavity. However, the molecular pathogenesis of HGS remains unclear. This study aimed to identify key pathogenic genes and explore the underlying molecular mechanisms of HGS using bioinformatics analysis and biological experiments.

**Methods:** Two datasets were downloaded from the Gene Expression Omnibus databases to find differentially expressed genes (DEGs) between HGS and normal tissue samples. Gene Ontology and Kyoto Encyclopedia of Genes and Genomes pathway enrichment analyses were applied to investigate the primary functions of the DEGs. The protein-protein interaction network of the DEGs was constructed, and the interactions of various genes were ranked.

**Results:** Topoisomerase IIα (TOP2A) was identified as the hub gene associated with survival and mutation. Gene Set Enrichment Analysis and Gene Set Variation Analysis were conducted to predict the potential biological functions of TOP2A. Furthermore, the TOP2A expression level was significantly up-regulated in HGS cell lines, SKOV3 and HEY. Moreover, the proliferation, migration, and invasion capacities of SKOV3 and HEY cells were strongly suppressed after TOP2A knockdown. In addition, the levels of phosphorylated Smad2 and Smad3, the key members of the transforming growth factor-β (TGF-β)/Smad pathway that regulate HGS tumorigenesis, strongly decreased after knockdown of TOP2A.

**Conclusions:** This study identified that TOP2A was up-regulated in HGS, and it accelerated HGS progression via the TGF-β/Smad pathway. The findings provided a blueprint for TOP2A serving as a therapeutic target and a treatment response prediction biomarker for HGS.

## Introduction

High-grade serous ovarian cancer (HGS), one of the most lethal forms of gynecologic malignancies, accounts for 75% of ovarian carcinomas and 90% of all mortalities 1. Despite advances in therapeutic strategies, the 5-year survival rate is only 45% due to its confusing symptoms and lack of screening for early detection 2. To date, the molecular pathogenesis of HGS has not been elucidated. Hence, it is an urgent requirement to understand better the molecular mechanisms in HGS tumorigenesis and discover new therapeutic targets and diagnostic biomarkers.

In recent years, more potential biomarkers have been discovered based on the rapidly developing microarray and high-throughput sequencing technologies 3. A series of studies used free public gene expression data, such as Gene Expression Omnibus (GEO) and the Cancer Genome Atlas (TCGA) databases, and bioinformatics methods to identify genes associated with ovarian cancer (OC) progression^4^, ^5^. In our present study, two GEO databases were utilized to identify topoisomerase IIα (TOP2A) as the key pathogenic gene, and a variety of experimental assays were applied to understand its biological functions. TOP2A encodes DNA topoisomerase and regulates the topological state of DNA during transcription, replication, and repair 6, 7. Several studies demonstrated that TOP2A participated actively in carcinogenesis in a range of cancer types, including breast, endometrial, and colon; higher TOP2A expression was indicative of poor prognosis 8-10. However, the expression level and underlying role of TOP2A in tumorigenesis of HGS has not been elucidated yet, and a better understanding of this would be of great significance for the clinical diagnosis, treatment, and prevention of HGS.

The aim of our study was to indicate key pathogenic genes between HGS cancer and normal tissue samples, using a series of bioinformatics approaches. In the study, we identified the biological function of the hub gene in HGS. The study reported that TOP2A was overexpressed in HGS cell lines, associated with poor cancer-specific, progression-free survival. We also revealed that TOP2A regulated proliferation and invasion, and played a role associated with antiapoptosis in HGS through the transforming growth factor-β (TGF-β)/Smad pathway.

## Materials and Methods

### HGS and normal tissue datasets

Data retrieved from multiple researches were used for integrated analysis in this study, including data from GEO and TCGA databases. Publicly available RNA-seq data and corresponding clinical pathological information of HGS patients in TCGA were obtained from UCSC Xena. Clinical characteristics included age, race, pharmaceutical therapy, radiation therapy, atomic neoplasm, stage, intermediate dimension, grade and outcomes, and follow-up. The somatic mutation status for OC (workflow type: VarScan2 Variant Aggregation and Masking) was obtained from the TCGA website. The microarray datasets GSE105437 and GSE18520 were downloaded from the GEO database) and annotated according to the platform of Affymetrix HG-U133 Plus 2.0 (GPL570).

Raw microarray data were downloaded and normalized using the Robust Multi-array Average (RMA) method with “affy,” an R package, for missing values estimation, background correction, log2 transformation, quantile normalization, and data summarization. The dataset GSE105437 included five normal ovarian and seven wound tissue samples, and ten epithelial high-grade, stage III or IV invasive serous ovarian samples; GSE18520 contained ten normal ovarian samples and fifty three high-grade primary tumor samples.

### Identification of DEGs from the GEO database

The two datasets were merged to one dataset, and then key differentially expressed genes (DEGs) were identified using a batch normalization method via R/language (“sva” package, R version 3.34). p-value<0.01 and |logFC|>1.5 were regarded as the cutoff criteria. Gene Ontology (GO) and Kyoto encyclopedia of genes and genomes (KEGG) pathway annotation analyses were performed using DAVID 6.8. The ontology included three categories: biological process (BP), molecular function (MF), and cellular component (CC). A p-value<0.05 was identified as the cut-off criterion for this analysis.

### Hub gene validation

The Kaplan-Meier plotter database was used to estimate the prognosis of HGS in 1435 patients. The HGS patients were divided into two groups according to the expression of the gene (high vs. low expression). The Oncomine database was used to check the expression values of TOP2A in normal and different cancer groups. The somatic mutation status for HGS was analyzed with “GenvisR” package.

### Gene Set Enrichment Analysis (GSEA) and Gene Set Variation Analysis (GSVA)

To investigate the TOP2A-mediated biological parameters in HGS, GSEA was conducted by cluster Profiler package. Further, h.all.v6.2.symbols.gmt was downloaded from the Molecular Signatures Database as the annotated gene set. FDR<0.05 and p-value<0.05 were selected as the enriched terms. Three hundred and eight HGS patients in the TCGA dataset were separated into high-expression and low-expression groups according to the median value of TOP2A. GSVA was conducted by “gsva” package to further validate different biological procedures between the two groups.

### Cell culture and transfection siRNA

HGS cell lines, including OVCAR3, SKOV-3, HEY, and CaoV3 and normal control cell line Hosepic (OSE) were purchased from ATCC. These cell lines were cultured in RPMI 1640 medium (Gibco, MD, USA) supplemented with 100 IU/ml penicillin, 100 μg/ml streptomycin (Gibco, MD, USA), and 10% fetal calf serum (HyClone, USA) in 5% CO_2_ at 37 °C. SKOV-3 and HEY cells were transfected with Lipofectamine™ RNAmax (Invitrogen, USA). TOP2A-target specific small interfering RNA (siRNA) was synthesized by JTS scientific (Beijing, China). The sense sequence of TOP2A-target-siRNA (si-TOP2A) was as follows: siRNA1, 5′-GGAAACAGCCAGUAGAGAATTUUCUCUACUGGCUGUUUCCTT-3′; siRNA2, 5′-GGUAACUCCUUGAAAGUAATTUUACUUUCAAGGAGUUACCTT-3′; siRNA3, 5′-GCUAUCAGCCUGGCCUUUATTUAAAGGCCAGGCUGAUAGCTT-3′. The sense sequence of si-control was 5′-UUCUCCGAACGUGUCAGGUTTUCCAGGTCUAGTT-3′. HEY and SKOV3 cells were transfected with 100 nmol si-TOP2A or si-control. After 48h of transfection, alterations of TOP2A mRNA were evaluated by quantitative real-time polymerase chain reaction (qRT-PCR).

### Reverse transcription and qRT-PCR

Total RNA from cultured cells was extracted using the Kit (Invitrogen, Calsbad, CA). First-Strand cDNA was synthesized with 1 µg total RNA using a ReverTraAceqPCR RT kit (Toyobo, Shanghai, China). Each reaction was conducted with SYBR Premix EX Taq™ (TaKaRa, Dalian, China) using 1 µg of cDNA in a final volume of 20 µl. qRT-PCR was operated with an ABI 7500 Real-Time PCR system (Applied Biosystems, Foster City, USA). All experiments were repeated at least three times. The primer sequences used to amplify TOP2A were: 5′-ACCATTGCAGCCTGTAAATGA-3′ (forward) and 5′-GGGCGGAGCAAAATATGTTCC-3′ (reverse). The cycle number of threshold (CT) value of TOP2A was normalized to the glyceraldehyde-3-phosphate dehydrogenase (GAPDH) value. Relative gene expression was determined by the comparative 2^-ΔΔ^CT method.

### Western blot analysis

The collected cells were washed with PBS and then lysed with RIPA lysis buffer (Thermo Fisher Scientific, MA, USA) supplemented with a protease inhibitor cocktail (Roche, Basel, Switzerland). Total protein amount was measured with a bicinchoninic acid assay (Thermo Fisher Scientific), and 30 µg total lysate per sample was subjected to SDS-PAGE followed by immunodetection with the following primary antibodies: Snail (CST; 3879), N-cadherin (CST; 13116),Vimentin (CST; 5741), E-cadherin (CST;3195), GAPDH (CST;5174), Twist (CST;46702), and Smad2/3 Antibody Sampler Kit (CST; 12747). For detection, the corresponding HRP-linked secondary antibody (CST) and enhanced chemiluminescence (Pierce, USA) were added.

### Cell proliferation assay

For this, 1×10^3^ cells were incubated in 96-well plates, and the absorbance of each well was measured at 450 nm using Tecan Infinite M1000 PRO (Tecan, Switzerland) on days 1-4. Then, 10 µl of Cell Counting Kit-8 (CCK-8, Dojindo, Rockville, USA) solution was added to each well and incubated for 2 h to evaluate cell proliferation. The experiment was performed with four replicates.

### Wound healing assay

Cell migration was evaluated with a wound healing assay. For this, 1×10^6^ SKOV3 or HEY cells were seeded on 6-well plates for transfection. After siRNA-transfection for 24h, cells were scratched and washed with PBS. Further, 0.1% FBS was added to allow the cells to move into the gap. The cells were photographed at 0 and 72 h in several pre-marked spots. The experiment was performed with three replicates. The cell migration was quantified by image J software. The migration rate was statistically analyzed using t-test.

### Transwell chamber migration and invasion assay

The transwell migration and invasion assay was conducted in a 24-well plate transwell chamber system (Corning, Inc., NY, USA). For the migration assay, 2×10^4^ cells suspended in 50 μl of serum-free PRIM-1640 were placed in the upper chamber, while 600 μl PRIM-1640 containing 10% FBS was placed in the lower chamber. After incubation for 24h at 37˚C, the cells were removed using cotton swabs from the upper chamber, fixed with 4% PFA, and stained with 0.1% crystal violet. The migrated cell number was counted in five randomly chosen fields (×200) using a phase contrast microscope and statistically analyzed. For the invasion assay, 1.2×10^5^ cells were seeded on transwells coated with 50 μl Matrigel (1:4 dilution with 0.2% BSA) (Sigma-Aldrich, St. Louis, MO, USA). The chamber was incubated at 37˚C as previously described, for 48h. Then, the lower side of the chamber was fixed, stained, and photographed with a microscope.

## Results

### Enrichment analysis of two DEGs

**Figure 1:**
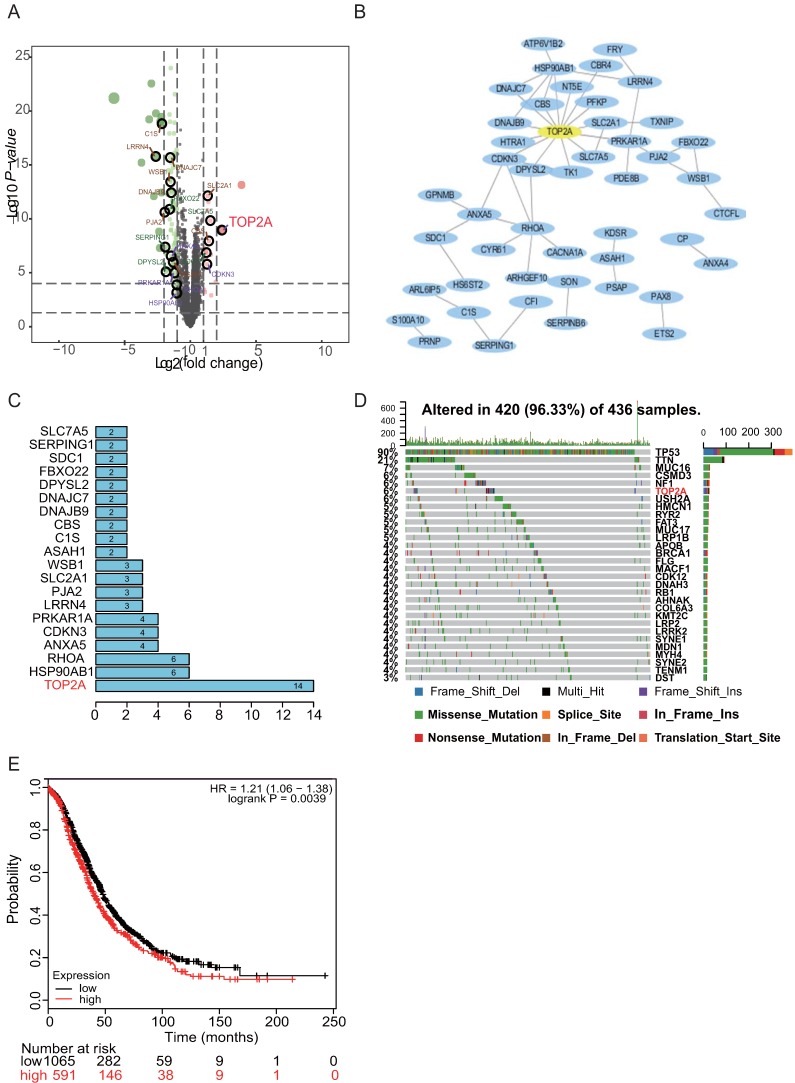
**Enrichment analysis of two DEGs and identification of the hub gene in HGS. (A)** Volcano plot of DEGs between HGS ovarian cancer and normal ovarian tissues. Red colored dots represent up-regulated DEGs and green colored dots represent down-regulated DEGs. **(B)** The PPI network of DEGs according to the inclusion criteria: combined score>0.4. **(C)** The barplot illustrating the ranks of protein-protein interactions. Ten candidate hub genes was are identified: TOP2A, HSP90AB1, RHOA, ANXA5, CDKN3, PRKAR1A, LRRN4, PJA2, SLC2A1, and WSB1. **(D)** TP53 and TOP2A are among the top six genes for somatic mutation burden for HGS. **(E)** TOP2A with significant survival in HGS.

The expression levels of the DEGs were mapped in two datasets, and the DEGs were clearly clustered into two groups: tumor samples and control samples. One hundred and fifteen DEGs were identified, out of which 17 were up-regulated and 98 were down-regulated in HGS (Figure 1A and Figure S1A). The heat map package was used to map the top 40 common up-regulated and down-regulated genes, as shown in Figure 1A. GO and KEGG analyses were performed to further analyze the biological functions and signal pathways of the DEGs in HGS. The bubble map was drawn using the ggplot2.R package for the top 32 significant biological processes based on p-value. The GO analysis results showed that up-regulated genes were particularly enriched in negative regulation of the apoptotic process, wound healing, etc. (Figure S1B).

### TOP2A as the hub gene screened from the PPI network and mutational events in HGS patients

The PPI network of the DEGs was constructed based on the information from the STRING database. After removing the separated and partially connected nodes; a complex DEG network was constructed. The barplot illustrated the ranks of protein-protein interactions, which showed that TOP2A interacted mostly with other proteins (Figure 1B). Subsequently, ten candidate hub genes were identified: TOP2A, HSP90AB1, RHOA, ANXA5, CDKN3, PRKAR1A, LRRN4, PJA2, SLC2A1, and WSB1 based on the rank sequence (Figure 1C).

Previous genetic profiling studies provided an accurate landscape of the driver gene mutations in HGS. Based on these facts, the mutational profiles of HGS were evaluated. The somatic mutation status for HGS was analyzed with “GenvisR” package. It was found that TP53 and TOP2A were among the top six genes for mutation burden, promoting HGS progression (Figure 1D). Then, the follow-up information of the ten hub genes in 1435 HGS patients was explored with the Kaplan-Meier plotter database. It was found that TOP2A, ANXA5, and LRRN4 were associated with ovarian cancer survival prognosis (Figure 1E and Figure S2). Hence, TOP2A was screened as the hub gene.

### Up-regulation of TOP2A in HGS tissues compared with normal ovarian tissues and cell lines

**Figure 2:**
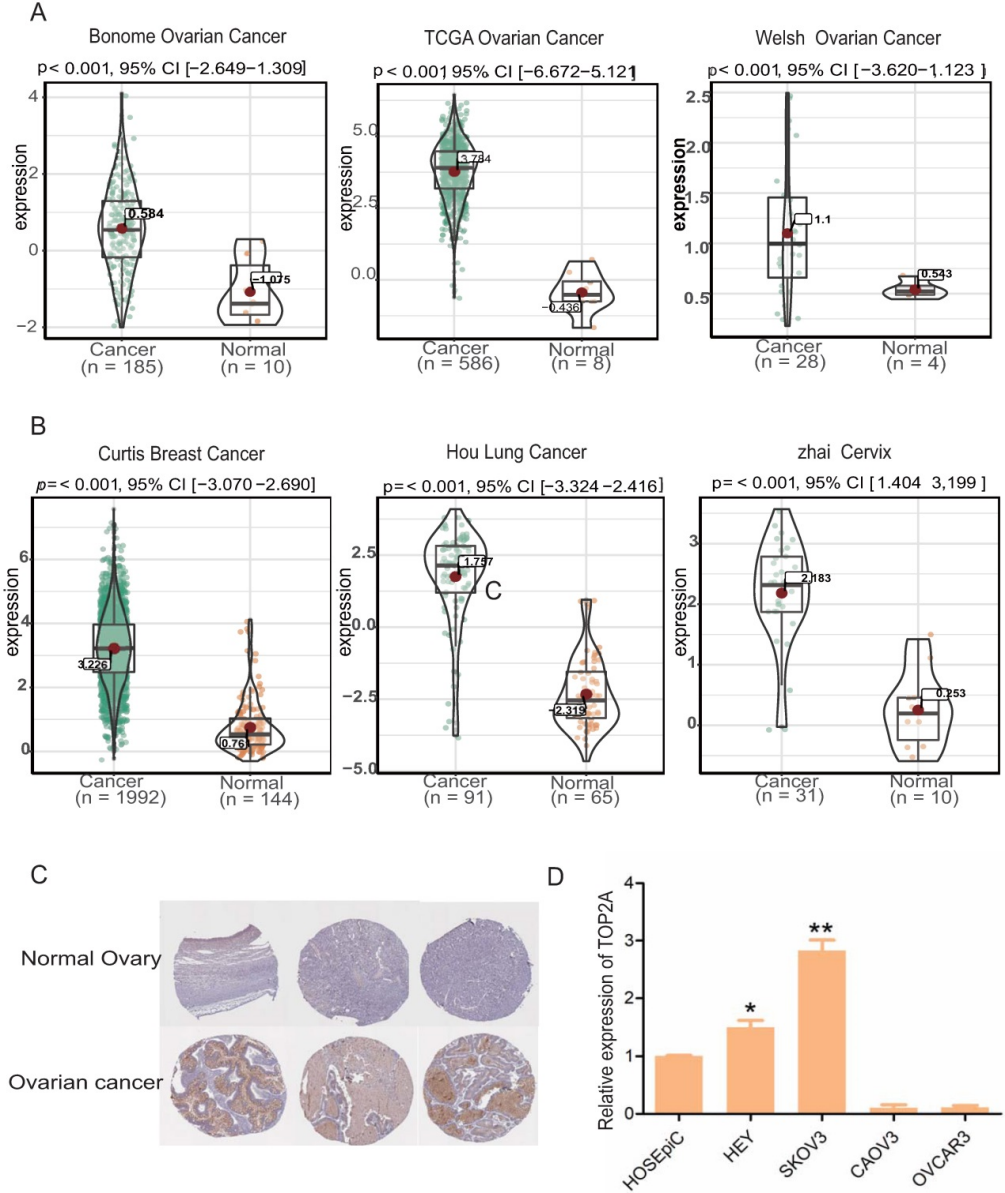
** Expression levels of TOP2A in multiple tumor types and ovarian cell lines. (A)** Expression level of TOP2A in HGS in oncomine datasets. **(B)** Expression levels of TOP2A in different cancers in oncomine datasets.** (C)** The representative protein expression levels of TOP2A in ovarian and normal tissues. Data were taken from the Human Protein Atlas database. **(D)** qRT-PCR analysis showing the gene expression levels of TOP2A in four kinds of ovarian cell lines. The GAPDH value is used as an internal control *p<0.05.

Oncomine database showed that TOP2A was significantly up-regulated at the transcriptional level in HGS tissues compared with normal ovarian tissues (Figure 2A). Further, the results of three other cancer studies, which included Curtis Breast Cancer, Hou Lung Cancer, and Zhai cervix, were combined. TOP2A was significantly up-regulated in the cancer samples and down-regulated in the normal samples (Figure 2B). In addition, immunohistochemistry staining revealed an increase in TOP2A protein level in the HGS tissues compared with normal tissues (www.proteinatlas.org) (Figure 2C). Furthermore, TOP2A also exhibited a significant up-regulation at the transcriptional level in the HGS cell lines SKOV3 and HEY compared with the normal ovarian cell line HOSEpic (p<0.05) (Figure 2D).

### Related biological pathways of TOP2A in ovarian cancer

**Figure 3:**
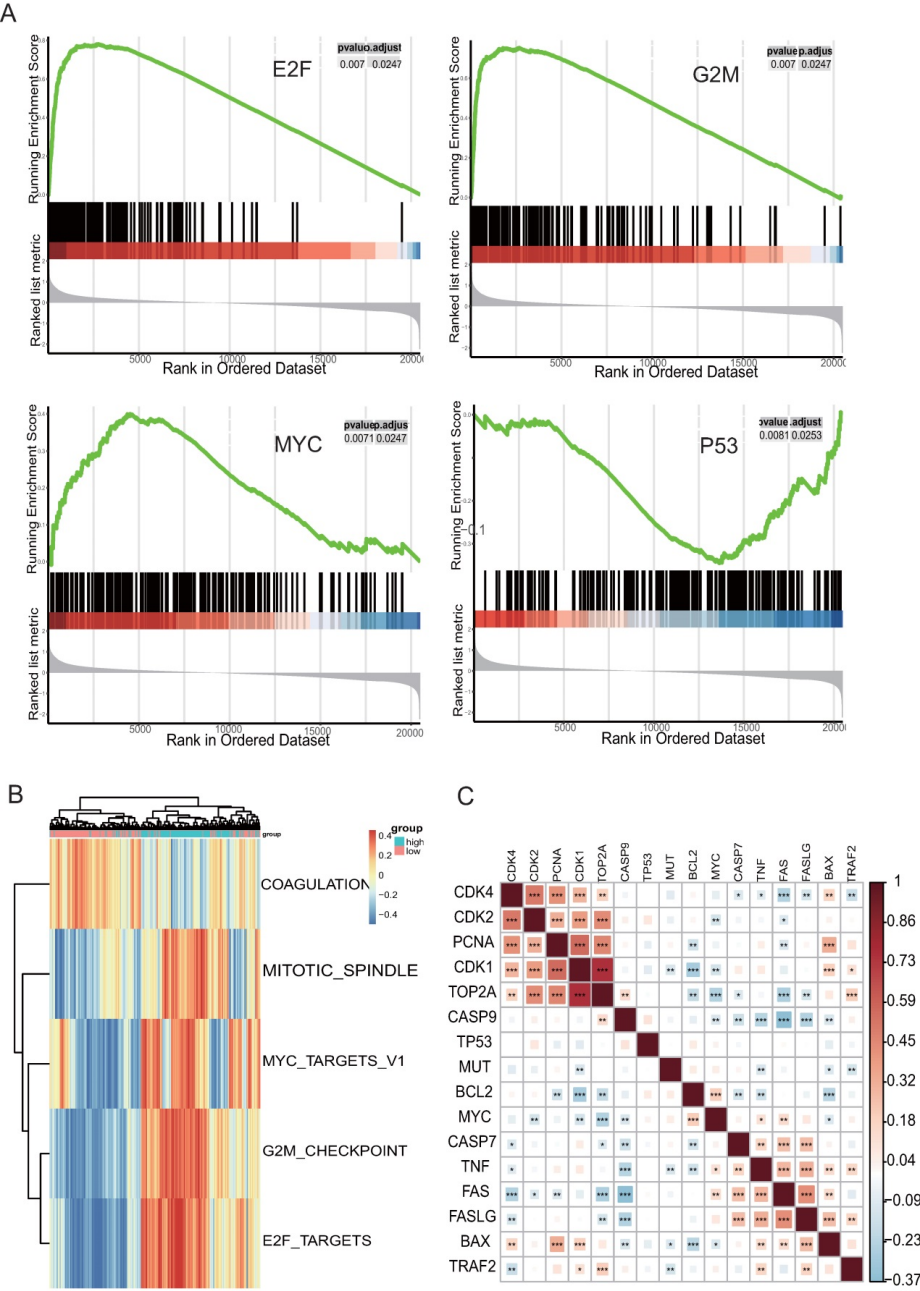
**Analysis of enriched pathways of TOP2A using GSVA and GSEA. (A)** Enriched pathway by the GSEA using MsigDB; G2M Checkpoint, E2F Targets, MYC Targets, and p53 pathway are enriched in HGS samples with highly expressed TOP2A. **(B)** Enriched pathway plot of GSVA. **(C)** Correlated heatmap illustrates that TOP2A is associated with genes related to the cell cycle, proliferation, and apoptosis.

Analysis of the potential mechanisms of TOP2A associated with HGS, GSEA, and GSVA with TCGA data was performed at the Hallmark level in two groups formed according to the median value of TOP2A. The three functional gene sets enriched were mainly associated with G2M Checkpoint, E2F and MYC Signaling, and one hallmark gene set of GSEA, “HALLMARK_P53_PATHWAY” (Figure 3A), which were validated by GSVA (Figure 3B). These results demonstrated that TOP2A was related to the cell cycle. The highly expressed TOP2A promoted cell proliferation but inhibited apoptosis. These biological pathways corresponded to the previous GO and KEGG pathway analysis. Correlated heatmap illustrated that TOP2A was associated with genes related to the cell cycle, proliferation, and apoptosis. Some crucial genes were associated with cell apoptosis and proliferation, such as *FADD*, *BCL2*, *CDK2*, *TP53*, *FOS*, and so forth. Their expression levels were obtained from TCGA datasets. Obviously, the proliferation related genes were clustered and positive for TOP2A, while the apoptosis associated genes were aggregated and negative for TOP2A (Figure 3C).

### Down-regulation of TOP2A restrained proliferation and accelerated apoptosis of HGS cell lines

**Figure 4:**
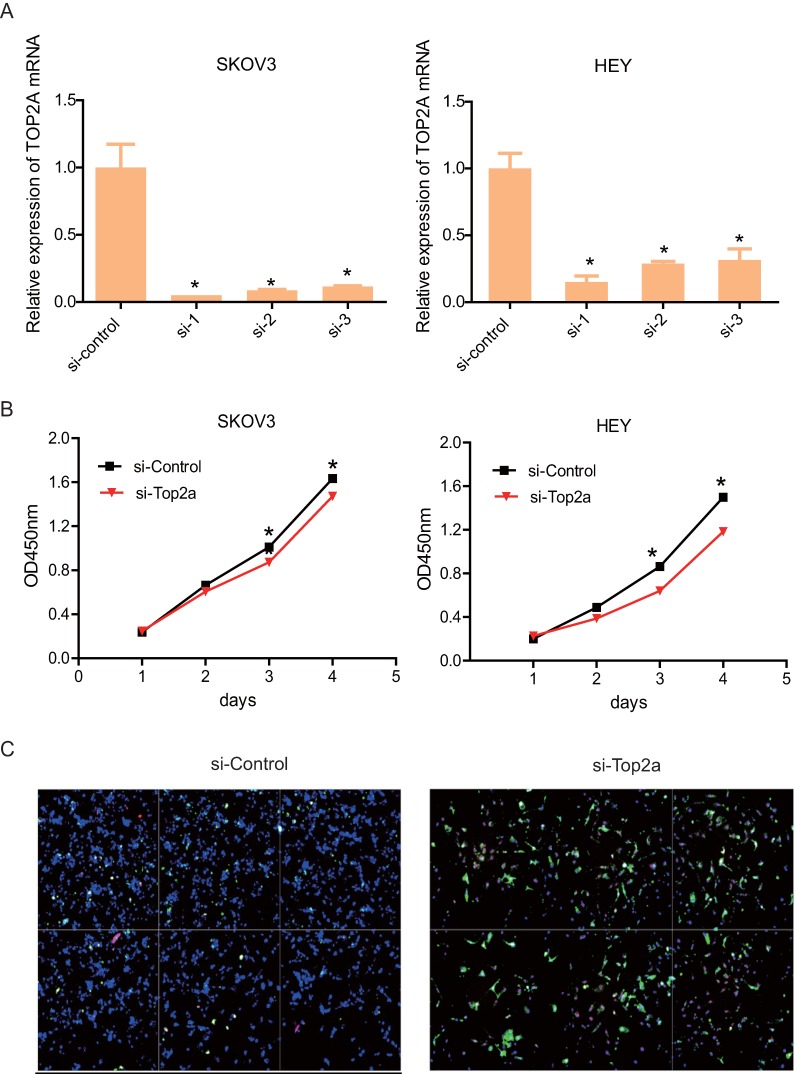
** Down-regulation of TOP2A represses HGS cell proliferation and promotes cell apoptosis. (A)** qRT-PCR validates efficiency by using distinct siRNA to knockdown TOP2A at the transcriptional level in SKOV3 and HEY cells. All values shown are mean ± SEM of triplicate measurements and repeated three times with similar results, *p<0.05 and **p<0.01. **(B)** The CCK8 proliferation assay is used to detect the viability of the two cell lines treated with si-control and si-TOP2A. All values shown are mean ± SEM of triplicate measurements and repeated three times with similar results, **p<0.01 and ***p<0.001. **(C)** Apoptosis of SKOV3 cells treated with si-control and si-TOP2A is detected using Annexin V (green) and 7-ADD (pink) staining. Nuclei are stained with DAPI (blue). The scale bar is 40 µm.

To detect the effect of TOP2A knockdown on cell viability and antiapoptosis ability, three distinct si-TOP2A were used to transfect SKOV3 and HEY cells. After 48h, the knockdown efficiency was validated by qRT-PCR (Figure 4A). The results showed that si-TOP2A 1 and 2 were more effective than si-TOP2A 3. Consequently, the cells that transfected si-TOP2A 1and 2 were co-transfected for subsequent assays. CCK8 assay suggested that knockdown of TOP2A restrained proliferation of SKOV3 and HEY cells drastically on the third day (Figure 4B). In addition, immunofluorescence staining showed that the si-TOP2A group considerably exhibited more apoptotic cells than the si-control group (Figure 4C).

### Down-regulation of TOP2A inhibited migration and invasion of HGS cells

**Figure 5:**
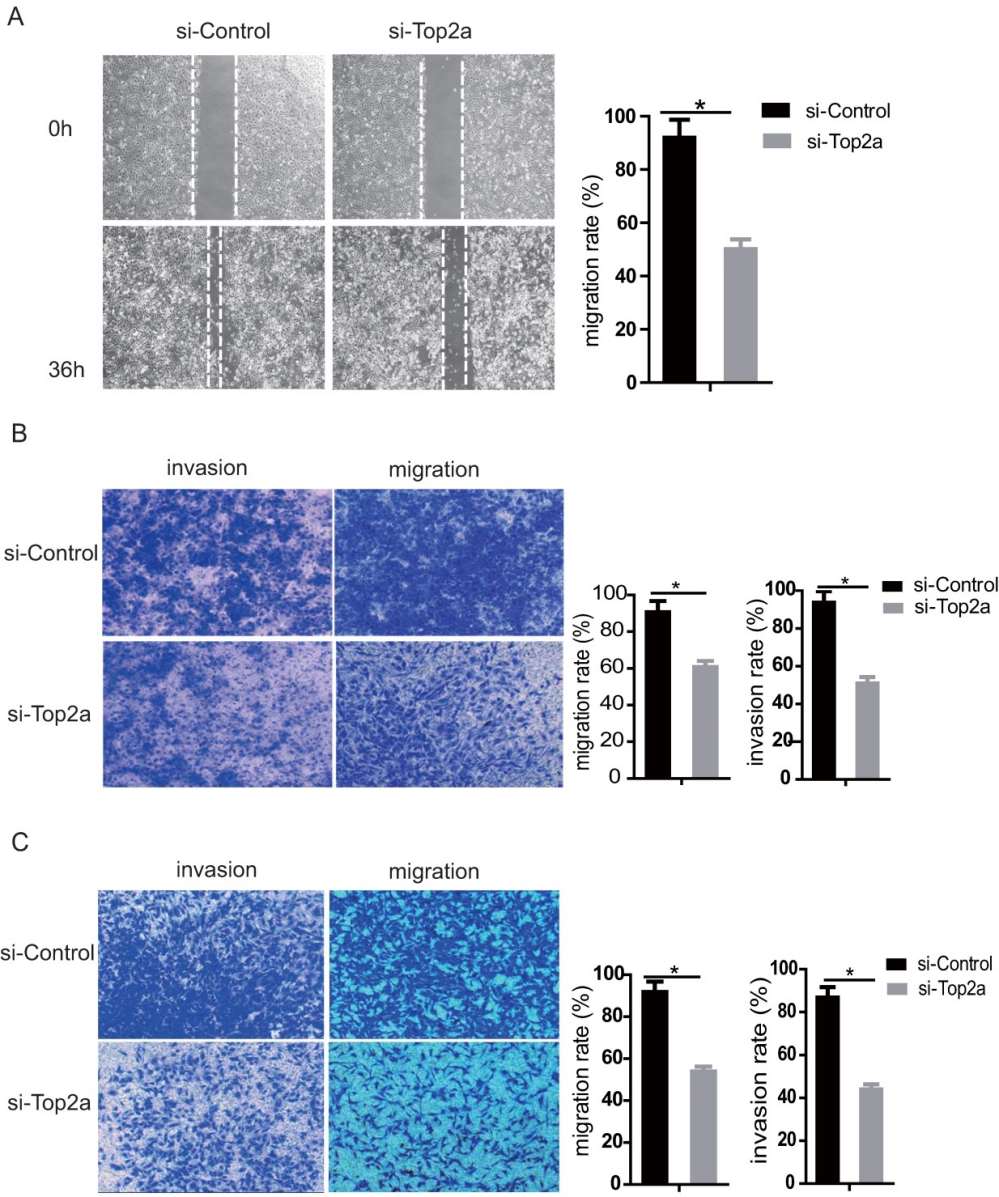
** Down-regulation of TOP2A represses the migration and invasion of HGS cell lines. (A)** The wound healing assay and statistical analysis after treatment of SKOV3 with si-control and si-TOP2A. **(B)** Transwell used for the migration and invasion assay with si-control and si-TOP2A treated SKOV3 cells. **(C)** Transwell used for the migration and invasion assay with si-control and si-TOP2A treated HEY cells. Scale bar for (B and C) is 50 µm. All values shown are mean ± SEM of triplicate measurements and repeated three times with similar results, *p<0.05 and **p<0.01.

The migration and invasion capacity of SKOV3 and HEY cells was strongly suppressed after TOP2A knockdown. The wound healing assay revealed that knockdown of TOP2A in SKOV3 cells remarkably reduced the number of migrated cells (p<0.05). The gap closure (%) was statistically analyzed (Figure 5A, right). Moreover, the transwell migration and invasion assay suggested that knockdown of TOP2A in SKOV3 (Figure 5B) and HEY (Figure 5C) cells reduced cell migration and invasion, which was confirmed by statistical analysis (p<0.05).

### Proteins involved in the TGF-β/Smad pathway and epithelial-mesenchymal transition (EMT) regulation were altered after TOP2A knockdown

**Figure 6:**
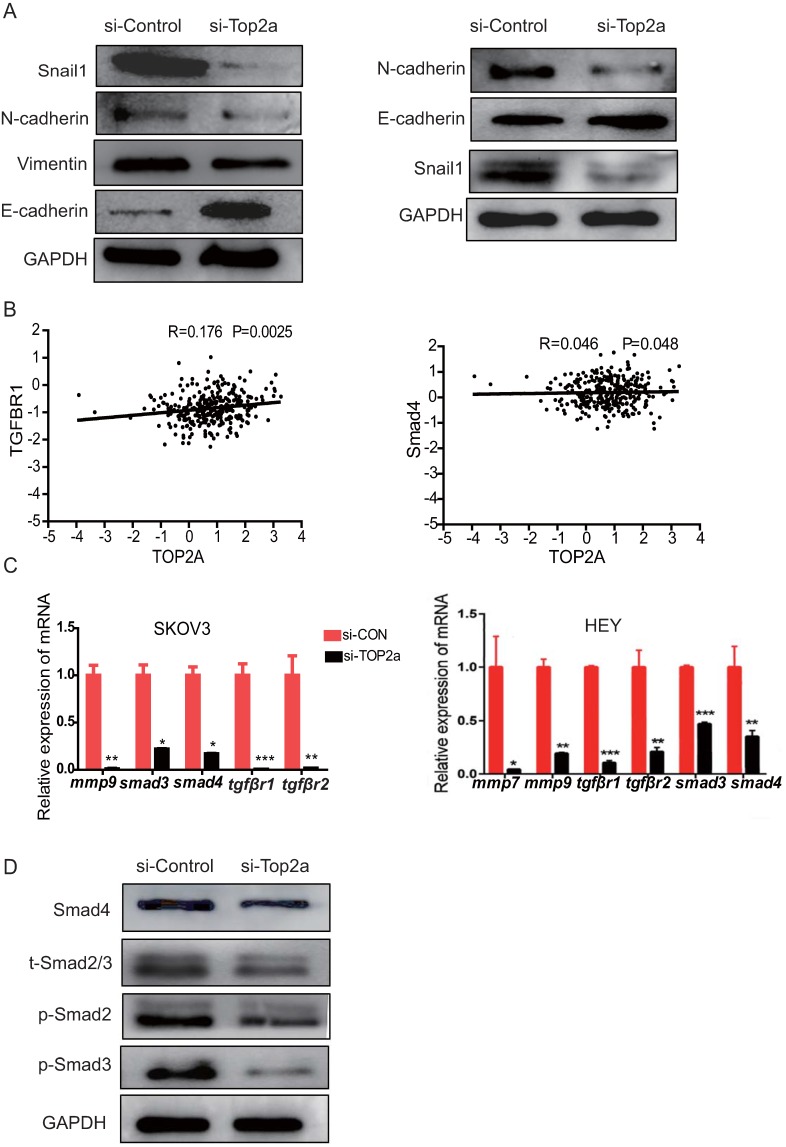
** Expression levels of proteins involved in EMT regulation and the TGF-β/Smad signaling pathway are altered after TOP2A knockdown. (A)** Western blotting analysis for proteins involved in EMT regulation reveals that E-cadherin is strongly increased, while N-cadherin, Vimentin, and Snail1 are considerably decreased after siRNA treatment. GAPDH is used as the loading control. **(B)** Correlation analysis among TOP2A, TGFβR1, and Smad4 in HGS. **(C)** qRT-PCR results show that Smad2/3, MMP9, and TGFβR1/2 are down-regulated in SKOV3 cells after TOP2A knockdown. **(D)** Western blot analysis for the proteins involved in the TGF-β/Smad signaling pathway reveals that p-Smad2, p-Smad3, and Smad4 are decreased in SKOV3 cells after siRNA treatment. GAPDH is used as the control.

To explore the mechanism of unregulated TOP2A promoted cell invasion and migration, proteins involved in the EMT process, including Snail1, N-cadherin, E-cadherin, and Vimentin, were detected using western blot analysis (Figure 6A). The data showed that the epithelial marker E-cadherin was upregulated and mesenchymal marker N- cadherin, Snail1, Vimentin were downregulated after TOP2A knockdown. Furthermore, the correlation between TOP2A and EMT related target pathway genes was explored, and the results revealed a moderate positive correlation among the expression levels of TOP2A, TGFβr1,and Smad4 (Figure 6B). Then, the expression levels of genes related to the TGF-β/Smad pathway, such as TGFBR1/2, SMAD2/3, MMP9, and so forth, were detected. The qRT-PCR results showed that Smad2/3, MMP9, TGFβr1, and TGFβr2 were down-regulated in the si-TOP2A-treated SKOV3 cells (Figure 6C). Moreover, the TGF-β/Smad pathway genes, including phosphorylated (p) Smad2 and Smad3, were affected in the si-TOP2A-treated SKOV3 cells (Figure 6D). TOP2A knockdown strongly suppressed the expression of Smad4, p-Smad2, and p-Smad3. These results suggested that TOP2A might accelerate the invasion and migration of HGS cells through the TGF-β/Smad pathway.

## Discussion

The mortality of patients with ovarian carcinomas is high among gynecologic malignant tumors. HGS accounts for 75% of ovarian carcinomas and 90% of all mortalities.^1^. Due to its confusing symptoms and lack of screening for early detection, HGS is still a major challenge for gynecologic and medical oncologists. To date, the molecular pathogenesis of HGS has not been elucidated. Therefore, exploring the molecular mechanisms and finding reliable tumor markers of HGS is of great significance for diagnosis and prognosis.

Gene expression profiling has successfully provided new insights into the molecular pathogenesis and classification of human malignancies 11-13. The bioinformatics methods were used in this study to integrate two datasets: GSE105437 and GSE18520. Through R package analysis, 115 common DEGs were identified; of these, 17 were up-regulated genes and 98 were down-regulated genes. Then, GO and KEGG pathway analyses were performed using DAVID. We found that the biological processes involved in the up-regulation of genes were mainly focused on cell apoptosis and proliferation. Transcriptional mapping indicated 10 aberrantly up-regulated candidates in HGS tissues. These included TOP2A, HSP90AB1, RHOA, ANXA5, CDKN3, PRKAR1A, LRRN4, PJA2, SLC2A, and WSB1. Previous genetic profiling studies provided an accurate landscape of the driver gene mutations in HGS 14, 15. TP53 and TOP2A were among the top six genes for mutation burden, promoting HGS progression. The PPI analysis showed that TOP2A was associated with the HGS patient's survival prognosis. To sum up, TOP2A was screened as the hub gene. However, in HGS, the oncogenic function and systematic investigation of TOP2A as a tumorigenic target has not been described previously.

The TOP2A gene encodes a DNA topoisomerase, an enzyme that controls and alters the topologic states of DNA during transcription. The expression of TOP2A has also been demonstrated in breast, colon, ovarian, and small cell lung cancers as a valuable prognostic marker for tumor advancements and recurrences, and as a predictor of poor survival. TOP2A overexpression in hepatocellular carcinoma correlates with early age onset, shorter patient survival, and chemoresistance. Furthermore, elevated expression of TOP2A mRNA is positively associated with high rate of recurrence and progression in primary non-muscle-invasive bladder cancer (n=103) 16.

Additionally, the TOP2A related pathway was predicted using GSEA and GSVA. The results showed that TOP2A was positive for G2M Checkpoint, E2F Targets, MYC Targets, and so forth, but negative for p53 Pathway. It was reported that TOP2A was up-regulated at the post-translational level, leading to TOP2A protein stabilization, inhibition of p53, and increased tumor-cell proliferation in lymphoblastic leukemia^17^.

Finally, the study explored the biological functions associated with TOP2A, revealing that TOP2A was involved in the cell cycle, proliferation, and apoptosis. The biological behavior of cancers is generally influenced by two major biological processes: proliferation and invasion. The correlated heatmap showed that TOP2A was positive with Smad2/3, MYC, MMP9, and so forth, indicating that TOP2A promoted cell proliferation, invasion, and migration via the TGF-β/Smad pathway. In our present study, we found that knockdown of TOP2A significantly inhibited the proliferation of HGS cells, revealing the essential role of TOP2A in cell proliferation. In addition, knockdown of TOP2A strongly suppressed the migration and invasion capacity of SKOV3 and HEY cells. Furthermore, the high content screening analysis suggested that TOP2A played a role in antiapoptosis. These results strengthened the evidence that TOP2A was involved in the progression of HGS. Previous studies demonstrated that TOP2A was overexpressed in adrenocortical carcinoma and influenced tumor progression, because knockdown of TOP2A in adrenocortical carcinoma cells decreased cell proliferation and invasion. Moreover, in liposarcoma, TOP2A was overexpressed, and knockdown of TOP2A reduced proliferation and invasion 18. Taken together, TOP2A was hence, proved to play a major role in proliferation and invasion. TGF-β regulated a fascinating array of cellular processes including cell proliferation, apoptosis, differentiation, migration, invasion, and adhesion. Most studies deemed that cancers progressed via the active TGF-β/Smad pathway. Our study demonstrated that TOP2A promoted cell proliferation, invasion, and migration by regulating the TGF-β/Smad pathway in HGS, which was unknown earlier.

## Conclusion

In our study, we identified TOP2A as the hub gene involved in the development of HGS, using different bioinformatics methods. Moreover, TOP2A accelerated HGS progression by regulating the TGF-β/Smad pathway. The distinct overexpression of TOP2A was further investigated for its potential clinical value in HGS.

## Supplementary Material

Supplementary figures.

## Abbreviations

TCGA: The Cancer Genome Atlas
GEO: Gene Expression Omnibus
DEGs: differentially expressed genes
GSEA: Gene Set Enrichment Analysis
GSVA: Gene Set Enrichment Variation Analysis
HGS: High-grade serous
OC: ovarian cancer
qRT-PCR: quantitative real-time polymerase chain reaction
Smad2/3/4: SMAD family member 2, 3, and 4
TGF-β: transforming growth factor-β
TOP2A: topoisomerase IIα
