# Supplementary material for: TOP2A Promotes Tumorigenesis of High-grade Serous Ovarian Cancer by Regulating the TGF-β/Smad Pathway
Source: J Cancer. 2020 Apr 25;11(14):4181–92. doi: 10.7150/jca.42736 (PMC7196274; doi:10.7150/jca.42736)
Supplement: Supplementary file 1 — Supplementary figures. [file jcav11p4181s1.pdf]

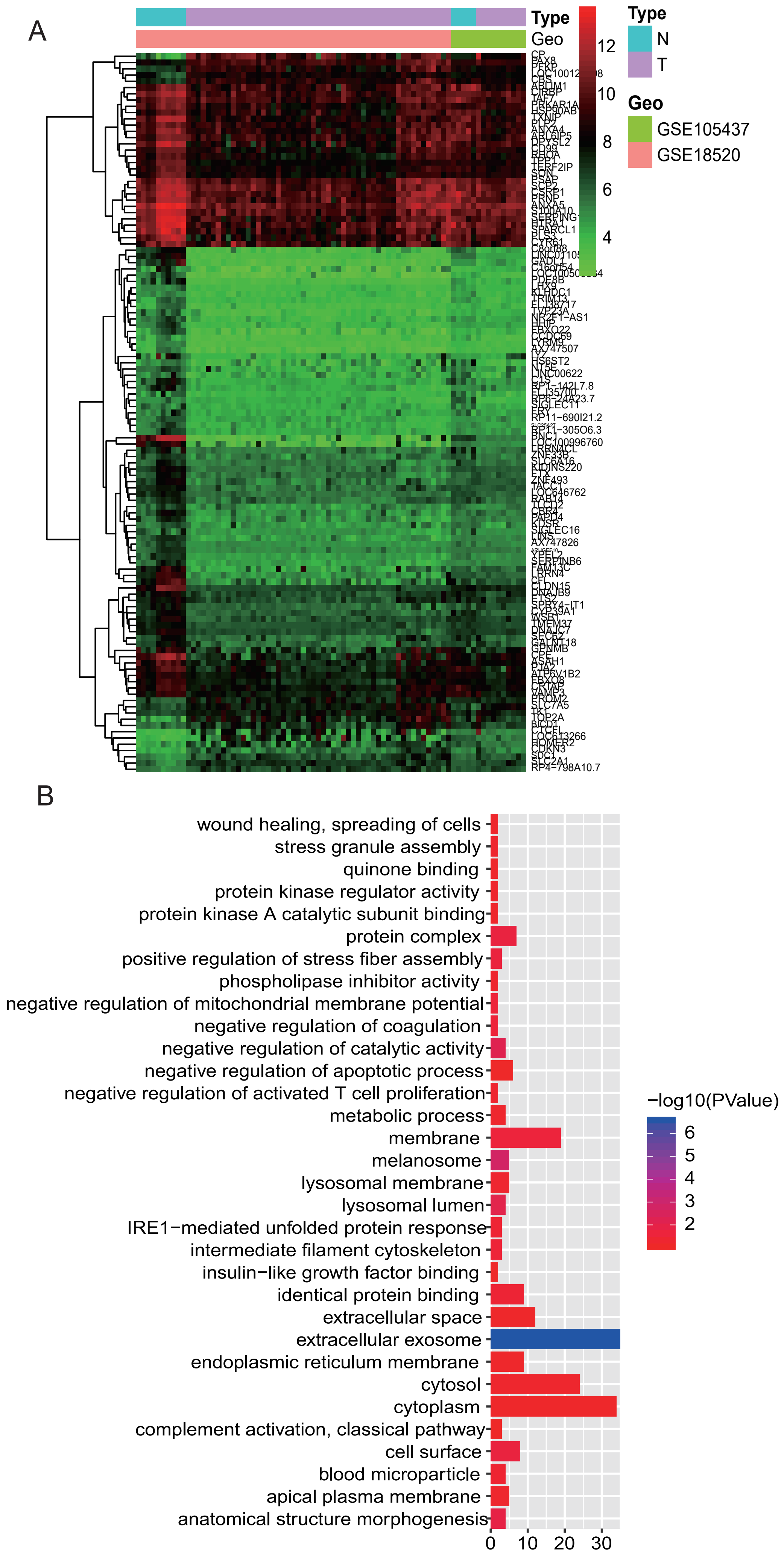
A
Type
Geo
12
10
8
6
4
Type
N
T
Geo
GSE105437
GSE18520
B
wound healing, spreading of cells
stress granule assembly
quinone binding
protein kinase regulator activity
protein kinase A catalytic subunit binding
protein complex
positive regulation of stress fiber assembly
phospholipase inhibitor activity
negative regulation of mitochondrial membrane potential
negative regulation of coagulation
negative regulation of catalytic activity
negative regulation of apoptotic process
negative regulation of activated T cell proliferation
metabolic process
membrane
melanosome
lysosomal membrane
lysosomal lumen
IRE1−mediated unfolded protein response
intermediate filament cytoskeleton
insulin−like growth factor binding
identical protein binding
extracellular space
extracellular exosome
endoplasmic reticulum membrane
cytosol
cytoplasm
complement activation, classical pathway
cell surface
blood microparticle
apical plasma membrane
anatomical structure morphogenesis
0
10
20
30
−log10(PValue)
6
5
4
3
2

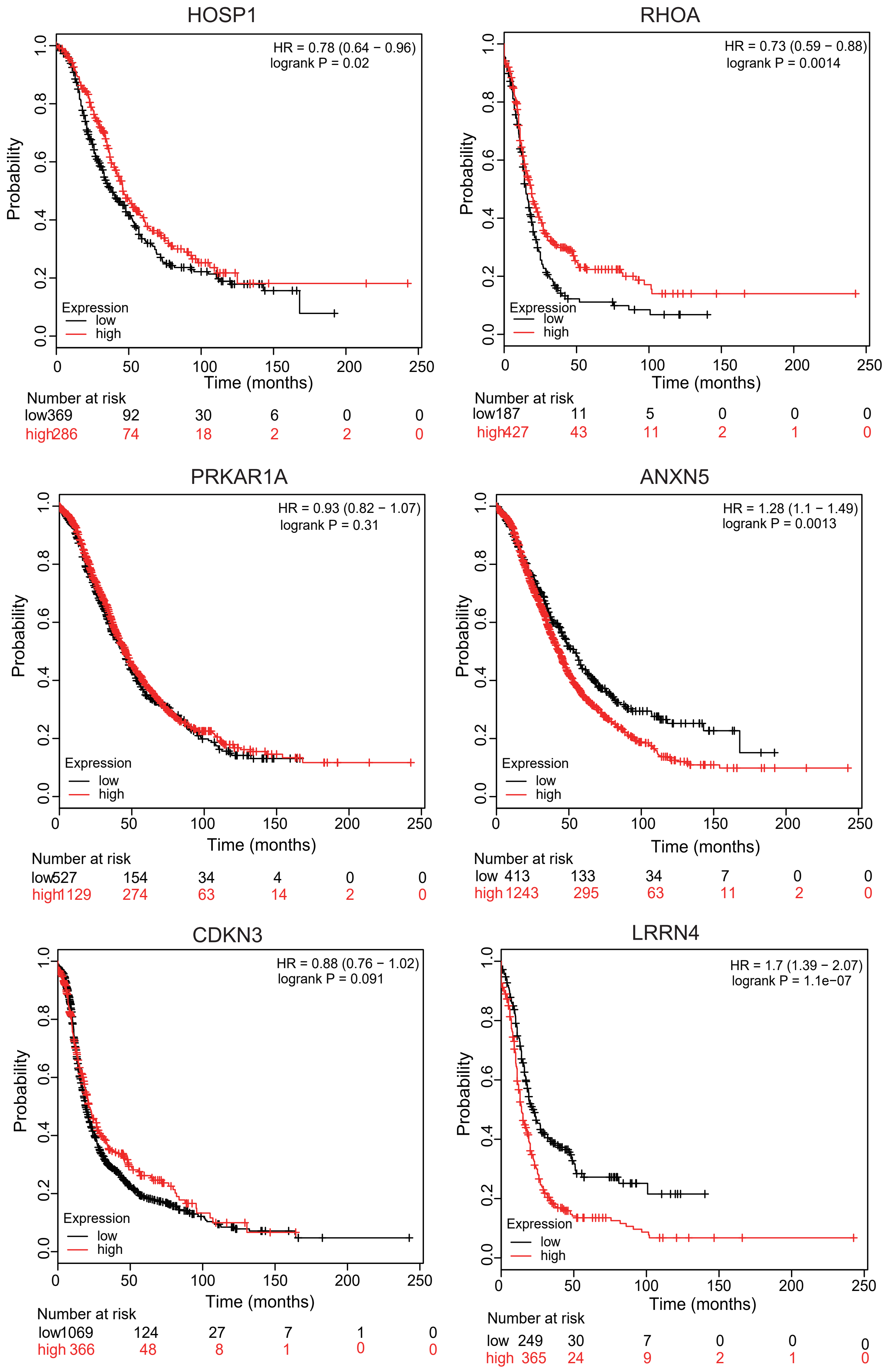

HOSP1
HR = 0.78 (0.64 − 0.96)
logrank P = 0.02
Probability
Time (months)
Expression
low
high
Number at risk
low 369 92 30 6 0 0
high 286 74 18 2 2 0
RHOA
HR = 0.73 (0.59 − 0.88)
logrank P = 0.0014
Probability
Time (months)
Expression
low
high
Number at risk
low 187 11 5 0 0 0
high 427 43 11 2 1 0
PRKAR1A
HR = 0.93 (0.82 − 1.07)
logrank P = 0.31
Probability
Time (months)
Expression
low
high
Number at risk
low 527 154 34 4 0 0
high 1129 274 63 14 2 0
ANXN5
HR = 1.28 (1.1 − 1.49)
logrank P = 0.0013
Probability
Time (months)
Expression
low
high
Number at risk
low 413 133 34 7 0 0
high 1243 295 63 11 2 0
CDKN3
HR = 0.88 (0.76 − 1.02)
logrank P = 0.091
Probability
Time (months)
Expression
low
high
Number at risk
low 1069 124 27 7 1 0
high 366 48 8 1 0 0
LRRN4
HR = 1.7 (1.39 − 2.07)
logrank P = 1.1e−07
Probability
Time (months)
Expression
low
high
Number at risk
low 249 30 7 0 0 0
high 365 24 9 2 1 0

Supplementary

Figure S1. (A) Heatmap of the 115 DEGs according to the selection criteria: $p < 0.05$ and $|logFC| > 2$.

(B) The Visualization of GO analysis of the DEGs.

Figure S2. Survival analyses of 6 candidate genes based on the Kaplan Meier Plotter database.
